# Supplementary material for: Novel Insights into Plant Genome Evolution and Adaptation as Revealed through Transposable Elements and Non-Coding RNAs in Conifers
Source: Genes (Basel). 2019 Mar 18;10(3):228. doi: 10.3390/genes10030228 (PMC6470726; doi:10.3390/genes10030228)
Supplement: Supplementary file 1 [file genes-10-00228-s001.pdf]

SUPPLEMENTAL INFORMATION

Novel insights into plant genome evolution and adaptation as revealed through transposable elements and non-coding RNAs in conifers

Yang LIU, Yousry A. EL-KASSABY

Department of Forest and Conservation Sciences, The University of British Columbia

2424 Main Mall, Vancouver, British Columbia V6T 1Z4 Canada

Table S1 Details of sequenced tree genomes (as of February 2019)

Species	Family	Common name	2*n*	References	URLs (genomes and other sequences)
Actinidia chinensis	Actinidiaceae	Kiwifruit	58	[1, 2]	http://bioinfo.bti.cornell.edu/cgi-bin/kiwi/download.cgi
Aquilaria agallocha	Thymelaeaceae	Agarwood	16	[3]	
Azadirachta indica	Meliaceae	Neem tree	30	[4]	
Betula nana	Betulaceae	Dwarf birch	28	[5]	http://www.birchgenome.org/data
Castanea mollissima	Fagaceae	Chinese chestnut	24	[6]	http://www.hardwoodgenomics.org/
Carica papaya	Caricaceae	Papaya	18	[7]	ftp://ftp.jgi-psf.org/pub/compgen/phytozome/v9.0/Cpapaya/assembly/Cpapaya_113.fa.gz
Citrus clementina	Rutaceae	Clementine mandarin	18	[8]	https://www.citrusgenomedb.org/organism/Citrus/clementina
Citrus sinensis	Rutaceae	Sweet orange	18	[8, 9]	http://citrus.hzau.edu.cn/orange/download/csi.chromosome.fa.tar.gz
Coffea canephora	Rubiaceae	Coffee	22	[10]	http://coffee-genome.org/coffeacanephora
Elaeis guineensis	Arecaceae	African oil palm	32	[11]	https://bioinformatics.psb.ugent.be/plaza/versions/plaza_v4_monocots/organism/view/Elaeis+guineensis
Eucalyptus camaldulensis	Myrtaceae	Red river gum	22	[12]	http://www.kazusa.or.jp/eucaly/
Eucalyptus grandis	Myrtaceae	Flooded gum	22	[13, 14]	https://eucgenie.org/
Fraxinus excelsior	Oleaceae	European ash	46	[15]	http://www.ashgenome.org/
Hevea brasiliensis	Euphorbiaceae	Pará rubber tree	36	[16-18]	http://www4a.biotec.or.th/rubber/
Jatropha curcas	Euphorbiaceae	Barbados nut	22	[19]	ftp://ftp.kazusa.or.jp/pub/jatropha/JAT_r4.5.genome.gz
Juglans regia	Fagaceae	Persian walnut	32	[20]	https://www.hardwoodgenomics.org/english-walnut-genome
Malus domestica	Rosaceae	Apple	34	[21, 22]	ftp://ftp.jgi-psf.org/pub/compgen/phytozome/v9.0/Mdomestica/assembly/Mdomestica_196.fa.gz

Morus notabilis	Moraceae	Mulberry	14	[23]	https://morus.swu.edu.cn/
Musa acuminate<br>*Musa balbisiana*	Musaceae	Banana (A/ B genome)	22	[24, 25]	http://www.promusa.org/Acuminata+genome
Olea europaea	Oleaceae	Olive tree	46	[26]	http://olivegenome.org/
Phoenix dactylifera	Arecaceae	Date palm	36	[27, 28]	https://www.genome.jp/kegg-bin/show_organism?org=pda
Picea abies	Pinaceae	Norway spruce	24	[29]	ftp://ftp.ncbi.nlm.nih.gov/genomes/all/GCA/900/067/695/
Picea glauca	Pinaceae	White spruce	24	[30, 31]	ftp://ftp.ncbi.nlm.nih.gov/genomes/all/GCA/000/411/955/
Pinus lambertiana	Pinaceae	Sugar pine	24	[32]	https://www.conifers.org/pi/Pinus_lambertiana.php
Pinus taeda	Pinaceae	Loblolly pine	24	[33-35]	ftp://ftp.ncbi.nlm.nih.gov/genomes/all/GCA/000/404/065/
Populus euphratica	Salicaceae	Desert poplar	38	[36]	ftp://ftp.ncbi.nih.gov/genomes/Populus_euphratica
Populus deltoides	Salicaceae	Eastern cottonwood	38		https://phytozome.jgi.doe.gov/pz/portal.html#!info?alias=Org_PdeltoidesWV94_er
Populus tremula	Salicaceae	European aspen	38	[37]	http://popgenie.org
Populus tremuloides	Salicaceae	Quaking aspen	38	[37]	http://popgenie.org
Populus trichocarpa	Salicaceae	Black cottonwood	38	[38]	ftp://ftp.jgi-psf.org/pub/JGI_data/phytozome/v7.0/Ptrichocarpa/assembly/Ptrichocarpa_156.fa.gz
Prunus mume	Rosaceae	Mei	16	[39]	https://www.genome.jp/kegg-bin/show_organism?org=pmum
Prunus persica	Rosaceae	Peach	16	[40]	ftp://ftp.jgi-psf.org/pub/JGI_data/phytozome/v7.0/Ppersica/assembly/Ppersica_139.fa.gz
Pyrus bretschneideri	Rosaceae	Chinese pear	34	[41]	https://bioinformatics.psb.ugent.be/plaza/versions/plaza_v4_dicots/organism/view/Pyrus+bretschneideri

Pyrus communis	Rosaceae	European pear	34	[42]	https://www.rosaceae.org/organism/Pyrus/communis
Quercus robur	Fagaceae	Pedunculate oak	24	[43]	https://www.hardwoodgenomics.org/organism/Quercus/robur
Rhizophora apiculata	Rhizophoraceae	Mangrove	36	[44]	http://evolution.sysu.edu.cn/Sequences.html
Salix purpurea	Salicaceae	Purple osier willow	38		https://phytozome.jgi.doe.gov/pz/portal.html#!info?alias=Org_Spurpurea
Salix suchowensis	Salicaceae	Purple willow	38	[45]	https://gold.jgi.doe.gov/project?id=96061
Theobroma cacao	Malvaceae	Cacao	20	[46, 47]	http://cocoagendb.cirad.fr/gbrowse/download/Theobroma_cacao_v1.0.pseudomolecule.fna.tar.gz
Vitis vinifera	Vitaceae	Grapevine	38	[48, 49]	ftp://ftp.jgi-psf.org/pub/compgen/phytozome/v9.0/Vvinifera/assembly/Vvinifera_145.fa.gz
Ziziphus jujuba	Rhamnaceae	Jujube	24	[50]	https://www.genome.jp/kegg-bin/show_organism?org=zju

Note S1 Source of botanical illustrations used in Figure 1

Abies, https://archive.org/details/traitdesarbres03moui/page/76; [51]

Cedrus, http://biodiversitylibrary.org/page/31681271#page/138/mode/1up

Juniperus, https://commons.wikimedia.org/wiki/File:Juniperus_communis_cones.jpg

Larix, https://commons.wikimedia.org/wiki/File:Illustration_Larix_decudua0.jpg

Picea, https://commons.wikimedia.org/wiki/File:Picea_abies_-_K%C3%B6hler%E2%80%93s_Medizinal-Pflanzen-105.jpg

Pinus, https://commons.wikimedia.org/wiki/File:Pinus_massoniana_SZ114.png

Pseudotsuga, http://archive.org/stream/traitdesarbres03moui#page/81/mode/1up; [51]

Thuja, https://commons.wikimedia.org/wiki/File:Thuja-orientalis.jpg

Tsuga, https://commons.wikimedia.org/wiki/Tsuga_canadensis

Supplementary references

1. Pilkington SM, Crowhurst R, Hilario E, Nardozza S, Fraser L, Peng Y, Gunaseelan K, Simpson R, Tahir J, Deroles SC *et al*: **A manually annotated *Actinidia chinensis* var. *chinensis* (kiwifruit) genome highlights the challenges associated with draft genomes and gene prediction in plants**. *BMC Genomics* 2018, **19**(1):257.
2. Huang S, Ding J, Deng D, Tang W, Sun H, Liu D, Zhang L, Niu X, Zhang X, Meng M *et al*: **Draft genome of the kiwifruit *Actinidia chinensis***. *Nature Communications* 2013, **4**:2640.
3. Chen CH, Kuo TC, Yang MH, Chien TY, Chu MJ, Huang LC, Chen CY, Lo HF, Jeng ST, Chen LF: **Identification of cucurbitacins and assembly of a draft genome for *Aquilaria agallocha***. *BMC Genomics* 2014, **15**:578.
4. Krishnan NM, Pattnaik S, Jain P, Gaur P, Choudhary R, Vaidyanathan S, Deepak S, Hariharan AK, Krishna PB, Nair J *et al*: **A draft of the genome and four transcriptomes of a medicinal and pesticidal angiosperm *Azadirachta indica***. *BMC Genomics* 2012, **13**:464.
5. Wang N, Thomson M, Bodles WJ, Crawford RM, Hunt HV, Featherstone AW, Pellicer J, Buggs RJ: **Genome sequence of dwarf birch (*Betula nana*) and cross-species RAD markers**. *Molecular Ecology* 2013, **22**(11):3098-3111.
6. Staton M, Zhebentyayeva T, Olukolu B, Fang GC, Nelson D, Carlson JE, Abbott AG: **Substantial genome synteny preservation among woody angiosperm species: comparative genomics of Chinese chestnut (*Castanea mollissima*) and plant reference genomes**. *BMC Genomics* 2015, **16**(1):744.
7. Ming R, Hou S, Feng Y, Yu Q, Dionne-Laporte A, Saw JH, Senin P, Wang W, Ly BV, Lewis KL *et al*: **The draft genome of the transgenic tropical fruit tree papaya (*Carica papaya* Linnaeus)**. *Nature* 2008, **452**(7190):991-996.
8. Wu GA, Prochnik S, Jenkins J, Salse J, Hellsten U, Murat F, Perrier X, Ruiz M, Scalabrin S, Terol J *et al*: **Sequencing of diverse mandarin, pummelo and orange genomes reveals complex history of admixture during citrus domestication**. *Nature Biotechnology* 2014, **32**(7):656-662.
9. Xu Q, Chen LL, Ruan X, Chen D, Zhu A, Chen C, Bertrand D, Jiao WB, Hao BH, Lyon MP *et al*: **The draft genome of sweet orange (*Citrus sinensis*)**. *Nature Genetics* 2013, **45**(1):59-66.
10. Denoeud F, Carretero-Paulet L, Dereeper A, Droc G, Guyot R, Pietrella M, Zheng C, Alberti A, Anthony F, Aprea G *et al*: **The coffee genome provides insight into the convergent evolution of caffeine biosynthesis**. *Science* 2014, **345**(6201):1181-1184.
11. Singh R, Ong-Abdullah M, Low ET, Manaf MA, Rosli R, Nookiah R, Ooi LC, Ooi SE, Chan KL, Halim MA *et al*: **Oil palm genome sequence reveals divergence of interfertile species in old and new worlds**. *Nature* 2013, **500**(7462):335-339.
12. Hirakawa H, Nakamura Y, Kaneko T, Isobe S, Sakai H, Kato T, Hibino T, Sasamoto S, Watanabe A, Yamada M *et al*: **Survey of the genetic information carried in the genome of *Eucalyptus camaldulensis***. *Plant Biotechnology* 2011, **28**(5):471-480.
13. Bartholome J, Mandrou E, Mabiala A, Jenkins J, Nabihoudine I, Klopp C, Schmutz J, Plomion C, Gion JM: **High-resolution genetic maps of *Eucalyptus* improve *Eucalyptus grandis* genome assembly**. *New Phytologist* 2015, **206**(4):1283-1296.
14. Myburg AA, Grattapaglia D, Tuskan GA, Hellsten U, Hayes RD, Grimwood J, Jenkins J, Lindquist E, Tice H, Bauer D *et al*: **The genome of *Eucalyptus grandis***. *Nature* 2014, **510**(7505):356-362.

15. Sollars ES, Harper AL, Kelly LJ, Sambles CM, Ramirez-Gonzalez RH, Swarbreck D, Kaithakottil G, Cooper ED, Uauy C, Havlickova L *et al*: **Genome sequence and genetic diversity of European ash trees**. *Nature* 2017, **541**(7636):212-216.
16. Lau N-S, Makita Y, Kawashima M, Taylor TD, Kondo S, Othman AS, Shu-Chien AC, Matsui M: **The rubber tree genome shows expansion of gene family associated with rubber biosynthesis**. *Scientific Reports* 2016, **6**:28594.
17. Rahman AY, Usharraj AO, Misra BB, Thottathil GP, Jayasekaran K, Feng Y, Hou S, Ong SY, Ng FL, Lee LS *et al*: **Draft genome sequence of the rubber tree *Hevea brasiliensis***. *BMC Genomics* 2013, **14**:75.
18. Tang C, Yang M, Fang Y, Luo Y, Gao S, Xiao X, An Z, Zhou B, Zhang B, Tan X *et al*: **The rubber tree genome reveals new insights into rubber production and species adaptation**. *Nature Plants* 2016, **2**:16073.
19. Sato S, Hirakawa H, Isobe S, Fukai E, Watanabe A, Kato M, Kawashima K, Minami C, Muraki A, Nakazaki N *et al*: **Sequence analysis of the genome of an oil-bearing tree, *Jatropha curcas* L.** *DNA Res* 2011, **18**(1):65-76.
20. Martínez-García PJ, Crepeau MW, Puiu D, Gonzalez-Ibeas D, Whalen J, Stevens KA, Paul R, Butterfield TS, Britton MT, Reagan RL *et al*: **The walnut (*Juglans regia*) genome sequence reveals diversity in genes coding for the biosynthesis of non-structural polyphenols**. *Plant Journal* 2016, **87**(5):507-532.
21. Li X, Kui L, Zhang J, Xie Y, Wang L, Yan Y, Wang N, Xu J, Li C, Wang W *et al*: **Improved hybrid de novo genome assembly of domesticated apple (*Malus* x *domestica*)**. *Gigascience* 2016, **5**(1):35.
22. Velasco R, Zharkikh A, Affourtit J, Dhingra A, Cestaro A, Kalyanaraman A, Fontana P, Bhatnagar SK, Troggio M, Pruss D *et al*: **The genome of the domesticated apple (*Malus* x *domestica* Borkh.)**. *Nature Genetics* 2010, **42**(10):833-839.
23. He N, Zhang C, Qi X, Zhao S, Tao Y, Yang G, Lee TH, Wang X, Cai Q, Li D *et al*: **Draft genome sequence of the mulberry tree *Morus notabilis***. *Nature Communications* 2013, **4**:2445.
24. D'Hont A, Denoeud F, Aury JM, Baurens FC, Carreel F, Garsmeur O, Noel B, Bocs S, Droc G, Rouard M *et al*: **The banana (*Musa acuminata*) genome and the evolution of monocotyledonous plants**. *Nature* 2012, **488**(7410):213-217.
25. Davey MW, Gudimella R, Harikrishna JA, Sin LW, Khalid N, Keulemans J: **A draft *Musa balbisiana* genome sequence for molecular genetics in polyploid, inter- and intra-specific Musa hybrids**. *BMC Genomics* 2013, **14**:683.
26. Barghini E, Natali L, Cossu RM, Giordani T, Pindo M, Cattonaro F, Scalabrin S, Velasco R, Morgante M, Cavallini A: **The peculiar landscape of repetitive sequences in the olive (*Olea europaea* L.) genome**. *Genome Biology and Evolution* 2014, **6**(4):776-791.
27. Al-Dous EK, George B, Al-Mahmoud ME, Al-Jaber MY, Wang H, Salameh YM, Al-Azwani EK, Chaluvadi S, Pontaroli AC, DeBarry J *et al*: ***De novo* genome sequencing and comparative genomics of date palm (*Phoenix dactylifera*)**. *Nature Biotechnology* 2011, **29**(6):521-527.
28. Al-Mssallem IS, Hu S, Zhang X, Lin Q, Liu W, Tan J, Yu X, Liu J, Pan L, Zhang T *et al*: **Genome sequence of the date palm *Phoenix dactylifera* L.** *Nature Communications* 2013, **4**:2274.
29. Nystedt B, Street NR, Wetterbom A, Zuccolo A, Lin YC, Scofield DG, Vezzi F, Delhomme N, Giacomello S, Alexeyenko A *et al*: **The Norway spruce genome sequence and conifer genome evolution**. *Nature* 2013, **497**(7451):579-584.

30. Birol I, Raymond A, Jackman SD, Pleasance S, Coope R, Taylor GA, Yuen MM, Keeling CI, Brand D, Vandervalk BP *et al*: **Assembling the 20 Gb white spruce (*Picea glauca*) genome from whole-genome shotgun sequencing data**. *Bioinformatics* 2013, **29**(12):1492-1497.
31. Warren RL, Keeling CI, Yuen MM, Raymond A, Taylor GA, Vandervalk BP, Mohamadi H, Paulino D, Chiu R, Jackman SD *et al*: **Improved white spruce (*Picea glauca*) genome assemblies and annotation of large gene families of conifer terpenoid and phenolic defense metabolism**. *The Plant Journal* 2015, **83**(2):189-212.
32. Stevens KA, Wegrzyn JL, Zimin A, Puiu D, Crepeau M, Cardeno C, Paul R, Gonzalez-Ibeas D, Koriabine M, Holtz-Morris AE *et al*: **Sequence of the sugar pine megagenome**. *Genetics* 2016, **204**(4):1613-1626.
33. Neale DB, Wegrzyn JL, Stevens KA, Zimin AV, Puiu D, Crepeau MW, Cardeno C, Koriabine M, Holtz-Morris AE, Liechty JD *et al*: **Decoding the massive genome of loblolly pine using haploid DNA and novel assembly strategies**. *Genome Biology* 2014, **15**(3):R59.
34. Wegrzyn JL, Liechty JD, Stevens KA, Wu LS, Loopstra CA, Vasquez-Gross HA, Dougherty WM, Lin BY, Zieve JJ, Martinez-Garcia PJ *et al*: **Unique features of the loblolly pine (*Pinus taeda* L.) megagenome revealed through sequence annotation**. *Genetics* 2014, **196**(3):891-909.
35. Zimin A, Stevens KA, Crepeau MW, Holtz-Morris A, Koriabine M, Marcais G, Puiu D, Roberts M, Wegrzyn JL, de Jong PJ *et al*: **Sequencing and assembly of the 22-Gb loblolly pine genome**. *Genetics* 2014, **196**(3):875-890.
36. Ma T, Wang J, Zhou G, Yue Z, Hu Q, Chen Y, Liu B, Qiu Q, Wang Z, Zhang J *et al*: **Genomic insights into salt adaptation in a desert poplar**. *Nature Communications* 2013, **4**:2797.
37. Sundell D, Mannapperuma C, Netotea S, Delhomme N, Lin Y-C, Sjödin A, Van de Peer Y, Jansson S, Hvidsten TR, Street NR: **The Plant Genome Integrative Explorer Resource: PlantGenIE.org**. *New Phytologist* 2015, **208**(4):1149-1156.
38. Tuskan GA, Difazio S, Jansson S, Bohlmann J, Grigoriev I, Hellsten U, Putnam N, Ralph S, Rombauts S, Salamov A *et al*: **The genome of black cottonwood, *Populus trichocarpa* (Torr. & Gray)**. *Science* 2006, **313**(5793):1596-1604.
39. Zhang Q, Chen W, Sun L, Zhao F, Huang B, Yang W, Tao Y, Wang J, Yuan Z, Fan G *et al*: **The genome of *Prunus mume***. *Nature Communications* 2012, **3**:1318.
40. Verde I, Abbott AG, Scalabrin S, Jung S, Shu S, Marroni F, Zhebentyayeva T, Dettori MT, Grimwood J, Cattonaro F *et al*: **The high-quality draft genome of peach (*Prunus persica*) identifies unique patterns of genetic diversity, domestication and genome evolution**. *Nature Genetics* 2013, **45**(5):487-494.
41. Wu J, Wang Z, Shi Z, Zhang S, Ming R, Zhu S, Khan MA, Tao S, Korban SS, Wang H *et al*: **The genome of the pear (*Pyrus bretschneideri* Rehd.)**. *Genome Research* 2013, **23**(2):396-408.
42. Chagné D, Crowhurst RN, Pindo M, Thrimawithana A, Deng C, Ireland H, Fiers M, Dzierzon H, Cestaro A, Fontana P *et al*: **The Draft Genome Sequence of European Pear (*Pyrus communis* L. 'Bartlett')**. *PLoS One* 2014, **9**(4):e92644.
43. Plomion C, Aury JM, Amselem J, Alaeitabar T, Barbe V, Belser C, Berges H, Bodenes C, Boudet N, Boury C *et al*: **Decoding the oak genome: Public release of sequence data, assembly, annotation and publication strategies**. *Molecular Ecology Resoures* 2016, **16**(1):254-265.
44. Consortium TIM, Lyu H, Li J, Yang M, Zhou R, Xu S, Guo W, Huang Y, Zhang Z, He Z *et al*: **The origin, diversification and adaptation of a major mangrove clade (Rhizophoreae) revealed by whole-genome sequencing**. *National Science Review* 2017, **4**(5):721-734.

45. Dai X, Hu Q, Cai Q, Feng K, Ye N, Tuskan GA, Milne R, Chen Y, Wan Z, Wang Z *et al*: **The willow genome and divergent evolution from poplar after the common genome duplication**. *Cell Research* 2014, **24**(10):1274-1277.
46. Argout X, Salse J, Aury JM, Guiltinan MJ, Droc G, Gouzy J, Allegre M, Chaparro C, Legavre T, Maximova SN *et al*: **The genome of *Theobroma cacao***. *Nature Genetics* 2011, **43**(2):101-108.
47. Motamayor JC, Mockaitis K, Schmutz J, Haiminen N, Iii DL, Cornejo O, Findley SD, Zheng P, Utro F, Royaert S *et al*: **The genome sequence of the most widely cultivated cacao type and its use to identify candidate genes regulating pod color**. *Genome Biology* 2013, **14**(6):r53.
48. Jaillon O, Aury JM, Noel B, Policriti A, Clepet C, Casagrande A, Choisne N, Aubourg S, Vitulo N, Jubin C *et al*: **The grapevine genome sequence suggests ancestral hexaploidization in major angiosperm phyla**. *Nature* 2007, **449**(7161):463-467.
49. Velasco R, Zharkikh A, Troggio M, Cartwright DA, Cestaro A, Pruss D, Pindo M, Fitzgerald LM, Vezzulli S, Reid J *et al*: **A high quality draft consensus sequence of the genome of a heterozygous grapevine variety**. *PLoS One* 2007, **2**(12):e1326.
50. Liu MJ, Zhao J, Cai QL, Liu GC, Wang JR, Zhao ZH, Liu P, Dai L, Yan G, Wang WJ *et al*: **The complex jujube genome provides insights into fruit tree biology**. *Nature Communications* 2014, **5**:5315.
51. Mouillefert P: **Traité des arbres & arbrisseaux forestiers, industriels et d'ornement cultivés ou exploités en Europe et plus particulièrement en France; donnant la description et l'utilisation de plus de 2400 espèces et 2000 variétés**. Paris: Librairie des sciences naturelles; 1892.
